# *Emaravirus*: A Novel Genus of Multipartite, Negative Strand RNA Plant Viruses 

**DOI:** 10.3390/v4091515

**Published:** 2012-09-12

**Authors:** Nicole Mielke-Ehret, Hans-Peter Mühlbach

**Affiliations:** Biocentre Klein Flottbek, University of Hamburg, Ohnhorststrasse 18, Hamburg 22609, Germany; Email: nicole_mielke@hotmail.com

**Keywords:** *European mountain ash ringspot-associated virus*, *Fig mosaic virus*, rose rosette virus, raspberry leaf blotch virus, pigeonpea sterility mosaic virus, High Plains virus

## Abstract

Ringspot symptoms in European mountain ash (*Sorbus aucuparia* L.), fig mosaic, rose rosette, raspberry leaf blotch, pigeonpea sterility mosaic (*Cajanus cajan*) and High Plains disease of maize and wheat were found to be associated with viruses that share several characteristics. They all have single-stranded multipartite RNA genomes of negative orientation. In some cases, double membrane-bound virus-like particles of 80 to 200 nm in diameter were found in infected tissue. Furthermore, at least five of these viruses were shown to be vectored by eriophyid mites. Sequences of *European mountain ash ringspot-associated virus* (EMARaV), *Fig mosaic virus* (FMV), rose rosette virus (RRV), raspberry leaf blotch virus (RLBV), pigeonpea sterility mosaic virus and High Plains virus strongly support their potential phylogenetic relationship. Therefore, after characterization of EMARaV, the novel genus *Emaravirus* was established, and FMV was the second virus species assigned to this genus. The recently sequenced RRV and RLBV are supposed to be additional members of this new group of plant RNA viruses.

## 1. Introduction

In the taxonomy of viruses, genome organization as well as nucleic acid and deduced amino acid sequence data are the main attributes to describe a virus family or genus, in addition to particle morphology, transmission and serological typing. Today, a total number of 2,284 viruses are described, which are grouped in 18 families and 17 unassigned genera [1]. However, there are still several putative virus species which have been known for a long time but have not yet been classified. This might be due to inadequate characterization or dissimilarity to other known viruses. Among this group of putative novel viruses, agents associated with the ringspot disease of European mountain ash (*Sorbus aucuparia* L.), fig mosaic disease (FMD), rose rosette disease (RRD), High Plains disease (HPD) of wheat and maize, pigeonpea sterility mosaic disease (PPSMD) and raspberry leaf blotch disorder (RLBD) share similarities with respect to their genomic organization and some of their biological properties. They all have segmented genomes consisting of four or more negative sense RNAs, their putative virions are double membrane-bound particles, and most of the diseases were shown to be transmitted by eriophyid mites. After elucidation of the genome sequence of the *European mountain ash ringspot associated virus* EMARaV [2], the unassigned genus *Emaravirus* was established [3]. Meanwhile, *Fig mosaic virus* FMV [4,5,6] has been assigned to the genus *Emaravirus *[7]; rose rosette virus (RRV) [8] as well as raspberry leaf blotch virus (RLBV) [9] might be the next additional members.

Although genomic sequence information is limited for High Plains virus (HPV, now referred to as maize red stripe virus—MRSV [10]) and pigeonpea sterility mosaic virus (PPSMV) [11], sequence similarities were found to EMARaV and to FMV, indicating a putative phylogenetic relationship [2,4].

This communication will resume the state of knowledge about these viruses and their associated diseases. It will give an idea about the virus characteristics as well as the relationship between each other, and to already described and classified plant viruses. Several aspects of the viruses in the group are summarized in Table 1.

**Table 1:** Structural and biological characteristics of *Emaravirus*-related viruses.

Virus Species	Particle Morphology	Vector (Putative)	Host Species
EMARaV	DMBs 80–120 nm	(*Phytoptus pyri*)	*Sorbus aucuparia*
FMV	DMBs 90–200 nm	*Aceria ficus*	*Ficus carica*
RRV	DMBs 120–150 nm	*Phyllocoptes fructiphilus*	*Rosa multiflora* cultivated hybrid roses
RLBV	indistinct filamentous bodies	*Phyllocoptes gracilis*	*Rubus spp*.
MRSV (HPV)	filamentous structures and enveloped particles 80–200 nm	*Aceria tosichella*	*Zea mays*, *Triticum aestivum*
PPSMV	filamentous structures and DMBs 100–150 nm	*Aceria cajani*	*Cajanus cajan*

## 2. Viruses and Associated Diseases

### 2.1. EMARaV — Ringspot Disease of European Mountain Ash (*Sorbus aucuparia* L.)

**Figure 1:**
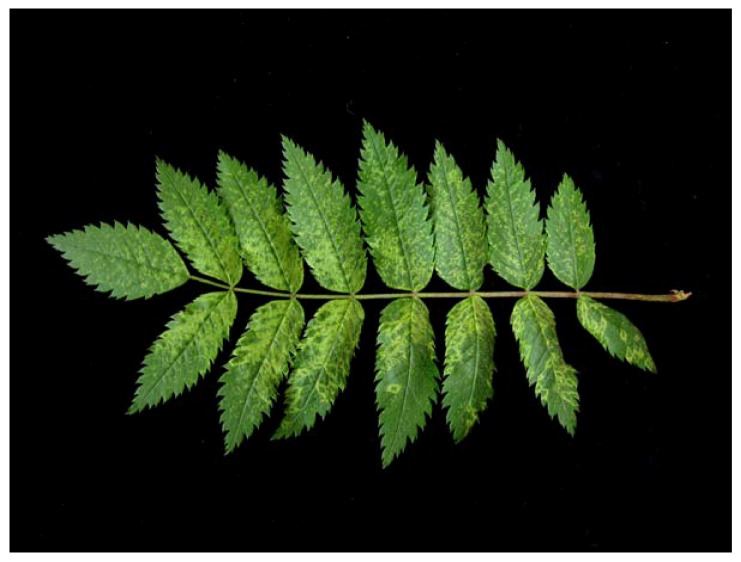
Typical symptoms of chlorotic ringspots and mottling on leaves of an EMARaV infected mountain ash tree.

Chlorotic ringspots and mottling have been described as typical symptoms of a disease affecting European mountain ash (*Sorbus aucuparia*), which was named ‘ringfleck mosaic’ or ‘ringspot disease’ [12]. Using nucleic acid-based techniques, EMARaV, the type member of the new genus *Emaravirus*, was found to be closely associated with the disease [2,13,14]. The two different symptoms, chlorotic mottling and ringspots, may occur separately or together, even on one single leaflet (Figure 1), and it was unclear for long time whether they are caused by the same pathogen. Recently it could be shown by quantitative reverse transcription (RT) PCR that in both areas of symptomatic leaves, the same amount of EMARaV-RNA was present [15]. Infected trees have often been found in clusters in many parts of Europe, the natural habitat of this species [16,17,18]. Close relatives of *S. aucuparia*, such as *S. aria* (whitebeam) and *S. torminalis* (service tree), have not been reported to be affected by ‘ringspot disease’ so far. Like many fruit tree species (e.g., apple, pear, plum) European mountain ash belongs to the family *Rosaceae*. Although the economical importance of *S*. *aucuparia* in fruit production is not that high, its ecological potential as a major pioneer tree species in reforestation projects, especially at erosion-endangered areas, is remarkable [19]. 

### 2.2. FMV — Fig Mosaic Disease (*Ficus carica* L.)

Fig mosaic disease (FMD) was first described in the 1930s in California [20]. Since then, many fig cultivars have been found to be susceptible to the infection [21] and FMD has been reported all around the world. Leaves of affected trees show mosaic or chlorotic spots. Furthermore, mottling, ringspots and leaf deformations can be observed [5,20,22,23]. Fruit may also show mosaic and in some cultivars premature fruit drop and reduced growth were reported [24]. Based on sequencing data and EM studies, more than seven different viruses were discussed in conjunction with FMD, including viruses in the genera *Potyvirus*, *Umbravirus*, *Luteovirus* and *Closterovirus *[21,25,26]. More recently, a novel, multipartite negative strand RNA virus, named *Fig mosaic virus* (FMV), and closely related to EMARaV, was identified [4,5,6]. A much more detailed description of FMD and the pathogens associated with the disease is given by Martelli [27]. 

### 2.3. RRV — Rose Rosette Disease *(Rosa spp.)*

Rose rosette disease (RRD), first described in the 1940s [28], is an indigenous disease in North America. The disease, alternatively called Witches’ broom of Rose, has been found in the central and eastern states of the USA on many cultivated hybrid roses as well as on a wide range of wild rose species [29]. Typical disease symptoms are described as rapid stem elongation, followed by breaking of axillary buds, leaflet deformation and wrinkling, bright red pigmentation, phyllody, and increased thorniness [29]. Among wild roses, *Rosa multiflora* is highly susceptible to the disease, being a putative source of infection of cultivated hybrid roses. During several decades in the last century *R. multiflora* was planted for hedges and wildlife improvement in rural areas as “living fences”, but extensive natural seeding and development of the rose plant could not be controlled by farmers. Therefore, it was declared a noxious weed, and as a biocontrol means for its eradication grafting of shoots of RRD infected plants onto plants in established *R. multiflora* stands (augmentation) was suggested [30]. The etiology of the disease remained enigmatic for long time, but the discovery of virus-like particles of 120–150 nm in diameter [31], the identification of the eriophyid mite *Phyllocoptes fructiphilus* as a vector of the disease [32], and the isolation of double-stranded RNA from infected material [33] indicated that a virus is associated with the disease. The recent characterization of four genomic RNAs of the rose rosette virus (RRV), a putative member of the genus *Emaravirus*, suggested that it is probably causing rose rosette [8].

### 2.4. RLBV — Raspberry Leaf Blotch Disorder *(Rubus spp.)*

Infestation of raspberry and other *Rubus* species by the raspberry leaf and bud mite *Phyllocoptes gracilis* (*Eriophyidae*) causes a complex disease syndrome called raspberry leaf blotch disorder (RLBD). It is characterized by yellow blotching as well as twisting of the leaves and distortion of leaf margins, decrease of overall plant growth by killing the terminal growing tip and reduction of fruit quality [34,35]. Although these symptoms resemble to some extent viral infections, convincing evidence for the association of a plant virus with RLBD had not been reported until recently. However, using molecular techniques, a new multipartite negative-strand RNA virus was discovered in plants showing RLBD symptoms, which was tentatively named raspberry leaf blotch virus (RLBV) [9]. Its genome organization and sequence similarities suggest that it is another member of the genus *Emaravirus*. 

### 2.5. MRSV (HPV) — High Plains Disease *(Zea mays, Triticum aestivum)*

High Plains disease (HPD) was first identified in 1993, where maize (*Zea mays*) and wheat (*Triticum aestivum*) with strong disease symptoms were observed in Idaho and Texas, and shortly after also in Kansas and Colorado (all USA) [36], but is probably the same disease as wheat spot mosaic, reported in the 1950s in the United States [37]. In the beginning, maize plants were tested positive for *Wheat streak mosaic virus *(WSMV) (*Potyvirus*) infection, but symptoms have been exceptionally strong, leading to the assumption that a second virus might be involved in the disease [38]. This second pathogen was tentatively named High Plains virus (HPV). Indeed, mixed infections of HPV and WSMV in wheat and maize are common and result in more severe symptoms [39]. Meanwhile, based on more detailed molecular studies, the name maize red stripe virus (MRSV) was suggested for the causative agent of HPD [10], which will be used invariably in the following paragraphs. 

In maize, disease symptoms are multifold. First evidence of infection becomes visible in young plants at 30 to 45 cm height [38]. Diseased maize plants show stunting, chlorosis (mosaic or streaking) and reddening which may lead to necrosis from the tip down the leaf. Moreover, in 50%–60% of infected maize plants, no ears are produced or ears contain only rudimentary seed, while others show smaller ears with reduced seed. HPD affected wheat is characterized by chlorotic spots or mosaic on the leaves and even complete yellowing of the plant. The virus also occurs in several other monocots, such as oat (*Avena sativa*), rye (*Secale cereale*), yellow foxtail (*Setaria glauca*), green foxtail (*Setaria viridis*) and downy brome (*Bromus tectorum*) [40]. As maize and wheat are two of the three main crops worldwide and the USA is the main producer for maize and the third largest for wheat, HPD causes serious economic damage in this country. However, the disease also reached other wheat and maize growing areas. First reports from Australia, South America (Brazil and Chile), Israel and possibly China indicate a progressive distribution of this novel disease [41,42].

### 2.6. PPSMV — Sterility Mosaic Disease of Pigeonpea *(Cajanus cajan)*

Sterility mosaic disease (SMD) of pigeonpea (*Cajanus cajan, Fabaceae*), also known as ‘Green Plague’, leads to reduced flowering, while vegetative growth is stimulated. Furthermore, SMD-affected plants show ringspots and mosaic. Leaf size may be reduced and, if existing, seeds are often small and deformed [43]. First reports of SMD in India and Myanmar were given as early as 1931 [44]. Today, the disease is distributed mainly on the Indian subcontinent (Nepal, India, Bangladesh) and in Myanmar. From the Eastern coast of Africa, where pigeonpea has been cultivated since the 1960s [45], and the Caribbean, another pigeonpea producer, no PPSMV-infection has been reported so far. Next to the crop *C. cajan*, its wild relatives, as e.g., *C. scarabaeoides*, can also be affected by SMD, supporting a spread of the ‘Green Plague’ [46].

Pigeonpea is one of the most important crop species in Southern Asia. It serves as main protein source for about 1.1 billion people. The seed contains 20%–30% of protein, including several essential amino acids, carbohydrates and vitamin A and C. Its stems are used for fuel and in handcraft, the leaves may serve as animal feed and the roots are able to nitrificate the soil and to release soil-bound phosphorus. In Nepal and India alone, yield losses of US$ 300 million due to SMD were already registered in 1993 and since then, infection has spread further [43]. PPSMV infection of plants younger than 45 days might result in a yield loss of 95%–100%; in the case of older pigeonpea plants, the losses vary between 26 and 97% [47]. Furthermore, SMD affected *C. cajan* is more susceptible to powdery mildew and spider mites [48,49], which increase the economical damage.

The detection of several RNA species and filamentous structures, as can be found in the genera *Tospovirus* and *Tenuivirus*, suggested that SMD is caused by a new RNA virus that was tentatively named pigeonpea sterility mosaic virus (PPSMV) [11]. Ultrathin leaf sections of infected *C. cajan* showed granular cell cytoplasm and deformation of the chloroplasts by large starch grains [50]. Today, several isolates of PPSMV differing in virulence are known. In addition, cultivars of *C. cajan* vary in susceptibility for PPSMV. Thus, PPSMV research is focused on identifying and producing resistant plants [46].

## 3. Virus Morphology

Four of the six virus diseases, ringspot disease of mountain ash, FMD, RRD and HPD, were shown to be associated with the occurrence of electron-dense structures known as double-membrane-bound bodies (DMBs) [51] with diameters differing within the range of 80 to 200 nm. These particles are supposed to be the virions (Table 1). 

**Figure 2:**
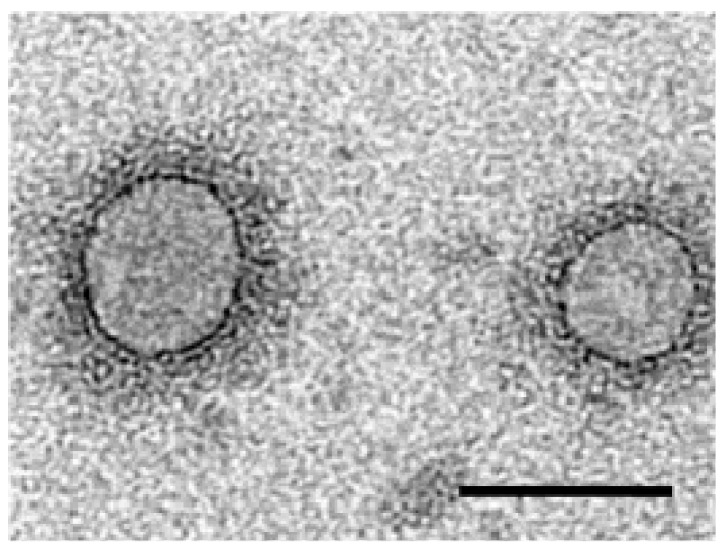
Double-membrane-bound bodies (DMBs) purified from EMARaV infected mountain ash leaves. Bar represents 100 nm.

In ultrathin sections of leaves from mountain ash trees affected by ringspot disease, DMBs of about 80–100 nm in diameter were detected in the cytoplasm [52], suggesting infection by tospoviruses, but further ELISA testing excluded the infection with *Tomato spotted wilt virus* (TSWV). Recently, we found similar structures in partially purified fractions from EMARaV-infected mountain ash leaves [3], obtained with a protocol developed for isolating TSWV virions [53] (Figure 2). 

DMBs resembling large tospoviral particles were also observed in infected fig tissues. The first DMBs in fig trees affected by fig mosaic were detected in 1970 [51]. Further studies supported the association of the disease with virus-like particles (VLPs), which vary in size, mainly between 120 and 160 nm in diameter [21,22,54,55]. 

Unusually large VLPs 120–150 nm in diameter were also found to be present in rose rosette affected tissue with typical disease symptoms [31]. In the same way as DMBs associated with infection of FMV and EMARaV [22,52], these putative RRV particles were surrounded by a double-membrane wall. 

Sections of PPSMV-infected *C. cajan* and *Nicotiana benthamiana* plants indicated the presence of 100–150 nm DMBs and fibrous inclusions, various in size and adjacent to the nucleus [43]. Numerous DMBs could be found in all cell types of the leaf except in phloem and bundle sheath parenchyma cells, where no or just a few particles have been observed. These DMBs could be decorated with antibodies directed at a 32 kDa protein, the putative nucleocapsid (N) protein of PPSMV [50]. Higher decoration with this antiserum was observed for electron-dense material, which was also present in the cells. In contrast, fibrous inclusions adjacent to the nucleus, resembling that of tenuivirus-infected cells and which contain a viral non-capsid protein [56,57], did not react with the antiserum. No DMBs were detectable in leaf sap, which remains infectious for 15 minutes at most. Attempts at partial purification, based on a gradient centrifugation, did not result in an enrichment of these structures [11]. 

In electron microscopic studies of High Plains diseased wheat, maize and barley, DMBs of 80–200 nm in diameter have been observed [10,38,58,59]. They could be found in all cell types, also in vascular parenchyma cells. These VLPs have been observed in maize leaves as early as two days after inoculating with mites collected from infected plants [10] and, as described for PPSMV, they were associated with electron-dense, amorphous material in the cytoplasm. Here also, the VLPs as well as the associated amorphous inclusions could be immunogold-labeled with an antiserum directed against the putative N-protein [58]. The decoration was shown to be concentrated in the core of the DMBs, while the surrounding membranes remained unlabelled. Following a protocol for purification of tospoviral particles [60] it was possible to enrich these DMBs [10]. It was noticed that primarily damaged virions were decorated with gold particles, indicating that the N-protein might not be the main component of the double membrane, but rather a structural protein of enclosed nucleocapsids as shown for tospoviruses [61]. 

Often, DMBs of these novel viruses were found to be located near the ER and Golgi cisterns [50,58,62], indicating that particle morphogenesis might take place at these intracellular host membranes as described for tospoviruses [63]. In addition to the DMBs, partial purification of MRSV and PPSMV by gradient centrifugation delivered flexuous structures, 3–10 nm in diameter and of undefined length, resembling tospoviral or tenuiviral nucleocapsids [11,43,58]. MRSV associated structures were shown to be decorated by the N-protein specific antiserum [58]. Like DMBs, these putative nucleocapsids seemed to be essential for the virus infection cycle, as they have been observed in PPSMV purifications with plant material from different places in India, as well as from experimentally mite-infected *C. cajanus* and *N. benthamiana* [11].

For RLBV, no ultrastructural studies with infected raspberry leaf tissue were undertaken until now, but in extracts enriched for putative virus particles, indistinct filamentous bodies similar to those reported for PPSMV and MRSV were found by examination under the microscope [9].

## 4. Genome Organization and Viral Proteins

The multipartite genomes of these novel viruses consist of single stranded (ss), negative-sense RNA. Double-strand RNA (dsRNA) preparations from infected plant tissue, Northern blot analyses as well as nucleic acid purification from enriched virus particle or nucleocapsid fractions indicated the presence of up to seven or eight RNA species, sized between 1.1 and 8 kb. Table 2 summarizes the number of presently known RNA species for each virus and their coding capacities.

**Table 2:** Genome organization and putatively encoded proteins of *Emaravirus*-related viruses.

Virus Species	RNA 1	RNA 2	RNA 3	RNA 4	RNA 5	RNA 6
EMARaV	7040 ntP1: 266 kDaRdRp	2335 ntP2: 75 kDaGlycoproteinprecursor	1559 ntP3: 35 kDaNucleocapsid	1348 ntP4: 27 kDaunknown	-	-
FMV	7093 ntP1: 264 kDaRdRp	2252 ntP2: 73 kDaGlycoprotein precursor	1490 ntP3: 35 kDaNucleocapsid	1472 ntP4: 40.5 kDaunknown	1752 ntP5: 59 kDaunknown	1212 ntP6: 22 kDaunknown
RRV	7026 nt P1: 265 kDaRdRp	2220 ntP2: 74 kDaGlycoproteinprecursor	1544 ntP3: 36 kDaNucleocapsid	1541 ntP4: 41 kDaMP	-	-
RLBV	7062 ntP1: 269 kDaRdRp	2135 nt P2: 75 kDaGlycoprotein precursor	1365 ntP3: 32 kDaNucleocapsid	1675 ntP4: 42 kDaMP	1718 ntP5: 56 kDaunknown	-
MRSV ^a^	RNA-L 7–8 kbRdRp?	RNA-M (double band) 2.5/2 kb	RNA-S1.4 kb32 kDaNucleocapsid	?	?	?
PPSMV ^b^	6.8 kb?	2.7 kb?	2.1 kb?	1.6 kb?	1.4 kbP5: 32 kDaNucleocapsid	1.2 kb?

^a^: MRSV has probably more RNAs in the RNA-S complex [9]; ^b^: PPSMV may contain an additional RNA species RNA 7 with 1.1 kb, respectively [11].

### 4.1. EMARaV

**Figure 3:**
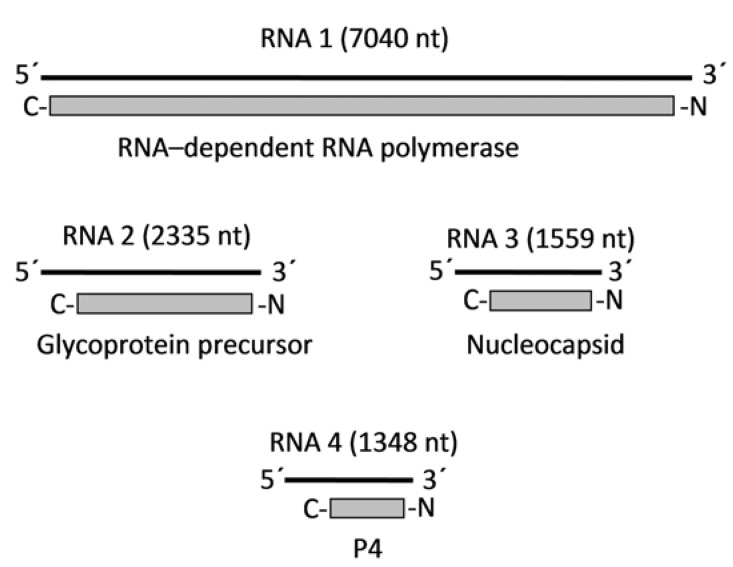
Genome organization of EMARaV. Virus genomic (minus-strand) RNAs are shown as black lines. Virus proteins encoded by the plus-strand mRNAs are shown as grey boxes.

EMARaV was the first virus among this group, for which full-length sequences of genomic RNAs were established [2]. Four RNA fragments could be detected and despite extensive search by dsRNA analyses, Northern blotting and PCR using primer pairs derived from the conserved RNA ends, no additional RNAs were found [2]. The four RNAs sequenced to date are sized between 1.3 and 7.0 kb and contain in the complementary form a single open reading frame (ORF) (Figure 3). The largest RNA 1 (7,040 nt) encodes the 266 kDa viral RNA-dependent RNA-polymerase (RdRp), which shows similarity to RdRps of bunyaviruses and tenuiviruses [2,13]. All conserved motifs (premotif A and motifs A-E) as well as a putative endonucleolytic center could be identified. The latter may be involved in the mechanism of cap snatching, which is typical for ss(-)RNA virus families, such as *Arenaviridae*, *Orthomyxoviridae* and *Bunyaviridae* [64,65] and recently demonstrated for FMV [66]. The second largest RNA (RNA 2 with 2235 nt) carries the gene for a 75 kDa glycoprotein precursor [2]. Sequence analyses revealed a conserved domain of the glycoprotein precursor of the vertebrate infecting genus *Phlebovirus* (*Bunyaviridae*) [67]. Furthermore, proteomics software predicted all essential elements as N-glycosylation sites, transmembrane helices, an N-terminal signal peptide and a putative processing site, where the precursor might be cleaved into two single glycoproteins of 52 and 23 kDa. Structural comparison with other glycoprotein precursors of bunyaviruses revealed a similar configuration [2]. RNA 3 (1559 nt) most likely encodes the N-protein (35 kDa), since a protein of that size was specifically enriched in a virus particle purification procedure with leaf material of EMARaV-infected mountain ash. The smallest RNA 4, with a size of 1,348 nt in total, contains the ORF for a 27 kDa protein (P4) of yet undetermined function. Although typical motifs of protein function have not been detected in the P4 of EMARaV, the P4 sequence of RRV contains motifs suggesting a function in cell-to-cell movement [8] and RLBV P4 contains a signal peptide at its N-terminus and was localized to the plasma membrane in transgenic tobacco [9]. These features will be discussed in detail in chapters 4.3 and 4.4, but the data suggest that P4 of EMARaV might exhibit a similar function. EMARaV RNAs have complementary ends, typical for ss(-)RNA viruses. Depending on the RNA species, complementarity spans over a stretch of 19 to 23 nt with short interruptions at two or four positions. Furthermore, the terminal 13 nt is fully conserved and shows a high consensus with the RNA ends of orthobunyaviruses and hantaviruses. These terminal structures of viral nucleic acids are discussed to be of protective function by forming the so-called panhandle structure and have regulatory capacity during encapsidation, replication and transcription [68,69,70]. 

### 4.2. FMV

Experimental strategies similar to the characterization of EMARaV allowed cDNA cloning and sequencing of six genomic RNAs of FMV [4,5,6,71,72]. Preparations of dsRNA from FMD-affected fig trees had revealed a changing number of molecules of up to 7 kb in length [6,24]. Initially, partial sequence information was obtained on two RNAs, indicating the relationship to viruses of the family *Bunyaviridae* and to EMARaV [6]. The genomic RNA molecules 1 to 4 of FMV exhibited some similarities to EMARaV with respect to the size and coding capacity of the individual RNAs. Each RNA species harbors in its complementary form (mRNA) one ORF and contains a highly conserved stretch of complementary nucleotides at the 3'- and 5'-end. Like EMARaV, RNA 1 encodes the viral RdRp, RNA 2 encodes a putative glycoprotein precursor, RNA 3 contains the gene of the nucleocapsid protein and RNA 4 encodes a protein of 40.5 kDa, which is larger than P4 of EMARaV, the function of which is also still unknown [4]. The newly-detected RNA-5 and RNA-6 contain two further proteins of unknown function [71,72]. FMV was assigned as new species *Fig mosaic virus* to the genus *Emaravirus *[7].

### 4.3. RRV

The strategy of dsRNA-based cDNA cloning and high throughput sequencing has recently allowed the characterizing of four genomic RNA segments of rose rosette virus (RRV) [8]. Genome organization and RNA sequences showed striking similarities to EMARaV and FMV, prompting the authors to conclude that it might be a further member of the genus *Emaravirus* [8]. As shown in Table 2, the four RNAs are quite similar in size to those of EMARaV and FMV and encode putative proteins with features similar to EMARaV and FMV orthologs. In addition, software-aided analyses predicted that P3, the putative nucleocapsid protein, can form homodimers and is bowl-shaped with interlocking protrusions and β-sheets, properties which allow the protein to bind RNA [8]. P4 derived from RNA 4 is still of undetermined function, but it contains an ATPase motif and a dnaK motif, the latter being known from dnaK-containing heat shock proteins. Since closteroviral Hsp70h was proven to be involved in virus cell-to-cell movement [73]; the authors suggested that RRV P4 might have a similar function. 

### 4.4. RLBV

The analysis of the genome organization of RLBV was started with random amplified cDNA derived from dsRNA [9]. A clone with some similarity to the nucleocapsid protein sequence of MRSV suggested that a new virus with similarities to the novel genus *Emaravirus* was present in raspberry plants exhibiting raspberry leaf blotch disorder. Therefore, RT-PCR with primers derived from the conserved terminal sequences of *Emaravirus* and stepwise using RNA-specific primers, followed by mass sequencing (Roche 454), led to characterization of five RNAs of the RLBV genome [9]. Four RNAs correspond to those found for other emaraviruses, but one was novel. RNA 1 and RNA 2 are very similar in size to the corresponding RNAs of the other emaraviruses (Table 2), and also encode the RdRp and a glycoprotein precursor, respectively. RNA 3, with 1,365 nt in size, is the shortest one of the five RNAs, but it encodes a putative nucleocapsid protein, similar to other emaraviruses, and is therefore called RNA 3, although RNA 4 and RNA 5 are larger. The similarity of the nucleocapsid sequence to other emaraviruses is varying; highest similarity (56%) was found to the nucleocapsid protein of MRSV, but only 44%–47% similarity was seen to EMARaV, RRV and FMV. The function of the putative 42 kDa protein P4, encoded by RNA 4, is still unknown, but the protein has some sequence similarities to the other unidentified proteins of emaraviruses. It contains a signal peptide at its N-terminus, and P4-GFP and P4-mRFP fusion proteins were localized to the plasma membrane and plasmodesmata in transgenic tobacco (*N. benthamiana and N. tabacum*) plants [9]. In addition, it co-localized with the tobacco mosaic virus 30K protein in plasmodesmata. Taken together, these findings led to the assumption that RLBV P4 could be involved in virus cell-to-cell movement. Up to now, P5 is unique to RLBV. It could be shown that the P5-GFP fusion protein was localized in aggregated structures in the cytoplasm, but its role in the life cycle of RLBV remains unknown [9].

### 4.5. MRSV

The RNA genome of MRSV is not fully characterized yet [10] (Table 2). Nucleic acid purifications from enriched virus preparations mainly delivered a large RNA-L of about 8 kb, a double band of 2.0 to 2.5 kb, called RNA-M, and a smaller fraction, called RNA-S, with an approximate size of 1.4 kb, which probably consists of multiple bands. By Northern blot-hybridization experiments with sense and antisense probes derived from RNA-S, viral genomic RNA was determined to be of negative polarity. Protein sequence predictions for RNA-L showed some similarities to the RdRp of viruses in the genus *Tospovirus. *While the predicted proteins encoded by the (probably) two different RNA species in the RNA-M complex showed no significant sequence similarities to proteins of other viruses, RNA-S has some similarities to EMARaV and PPSMV [2,3]. It is supposed to encode the 32 kDa nucleocapsid protein [10]. Partial virus purifications based on gradient centrifugation delivered predominantly a protein of 32 kDa, but sizes of 33 kDa have also been reported [10,38,74,75].

### 4.6. PPSMV

In contrast to the *Emaravirus* related virus species discussed above, the genome of PPSMV was reported to consist of seven or more RNAs [11]. Partial purification of virus-like particles, followed by nucleic acid preparation, revealed five to seven RNAs sized between 1.1 and 6.8 kb, although the largest RNA was not obtained in all purifications. The authors stated that the RNAs are of single stranded nature due to high sensitivity against RNase A even at high salt concentrations. Purified RNA was not infectious, indicating a possibly degradation during preparation or more likely the negative polarity of the viral genome. The main viral protein obtained during purification was supposed to be the N-protein and seems to be encoded by a 1.4 kb RNA named RNA 5. This protein differed in size, while Indian isolates from Andhra Pradesh (P) and Bangalore (B) possessed a 32 kDa N-protein, another isolate C from Tamil Nadu is characterized by a larger protein of about 35 kDa [43]. 

### 4.7. Sequence Similarities among All *Emaravirus* Related Viruses

Taken together, comparison of the so far published deduced amino acid (aa) sequences revealed remarkable similarities among EMARaV, FMV, RRV and RLBV as well as MRSV and PPSMV, but also to bunyaviruses and tenuiviruses. In particular, P1, the putative RdRp, shows significant conserved sections due to functional domains. Thus, the RdRps of EMARaV, FMV, RLBV and RRV were described as having aa identities of 49%–68% (56%–83% similarity) to their orthologs [2,4,5,6,8,9]. Unfortunately, no PPSMV or MRSV-specific RdRp sequences are available so far. The second protein P2 of these viruses, a putative glycoprotein precursor, also shows high similarities. The structural organization of EMARaV P2 was found to be very similar to the precursor molecules of bunyaviruses and tenuiviruses [2]. Nevertheless, their aa-sequences do not share any identity; only a short conserved motif of the phlebovirus glycoproteins (*Bunyaviridae*) has been identified, as was recently reported for RRV [8]. However, the putative glycoprotein precursors of EMARaV and FMV were reported to have aa-identities between 34% and 38% [4,5], while RRV P2 showed 51% aa identity with FMV and 40% with EMARaV [8] and RLBV has 46%–49% similarity to EMARaV, FMV and RRV [9]. Furthermore, for all six (putative) emaraviruses, major parts of the N-protein sequences (P3), are known and they show various similarities to each other [2,4,5,6,8,9,10,11]. Thus, FMV has highest similarity to EMARaV (49%) and PPSMV (42%), while identity with MRSV is only 27%. In contrast, RLBV shows 56% similarity to MRSV, but only 44%–47% to EMARaV, RRV and FMV. No similarity was found with members of the family *Bunyaviridae* or the genus *Tenuivirus*. The role of protein P4 could not be clarified for the *Emaravirus* related viruses under study, and there is no remarkable sequence similarity to proteins of other virus families or genera [2,4,8,9]. However, P4 of RRV shows 59% aa identity to the FMV ortholog [8], while P4 of RLBV has lower sequence similarities to EMARaV P4 (31%) and to FMV and RRV (41%) [9]. The size of P4 varies from 27 kDa (EMARaV) up to 42 kDa (RLBV). Since the predicted amino acid sequence for RRV P4 indicates an ATPase motif and a dnaK motif, a role of P4 in virus cell-to-cell movement is discussed [8]. This hypothesis is in agreement with the finding that P4 of RLBV contains an N-terminal signal peptide sequence and localized to the plasma membrane [9]. However, it is also conceivable that P4 proteins of the novel viruses have individual functions, which await further investigations. 

Furthermore, recent studies showed that the complementary RNA termini, characterized so far, are more or less identical among EMARaV, FMV, RRV, and RLBV (5'-AGUAGUGUUCUCC … GGAGUUCACUACU-3'). The sequence of the only available MRSV RNA terminus is also similar. High similarity was also found with the RNA ends of the genera *Orthobunyavirus* and *Hantavirus *within the family *Bunyaviridae*, but not with that of the plant pathogenic tospo- and tenuiviruses [2,4]. For PPSMV, no terminus sequences are available in the databases up to now.

## 5. Serological Relationship and Diagnostic Procedures

As particle and nucleocapsid morphology of EMARaV, PPSMV, MRSV and FMV resembled to some extent tospoviruses and tenuiviruses, efforts were made to detect these viruses with antisera against the two genera. But no reactions were obtained, indicating a low serological relationship, which can be easily explained by the recently published sequence comparison with viruses of these genera. Today, for the specific detection of identified proteins of this group of novel viruses, several antisera are available. However, the two antisera raised against the putative glycoproteins and the N-protein (P3) of EMARaV were not recommended for routine testing, due to weak reaction and background signals in Western blot with crude protein extracts [2,14]. But when the antiserum directed against P3 was used with partially purified virus fractions, a strong serological reaction was obtained with the 35 kDa nucleocapsid protein. The antisera for PPSMV and MRSV, directed against the 32 kDa N-proteins, are routinely used in ELISA testing and Western blot analyses [10,43,46,50,59,76,77,78]. The PPSMV-specific antiserum is able to detect different Indian isolates, although the reaction with the isolate C from Tamil Nadu is weaker than with the isolates P (Andhra Pradesh) and B (Bangalore) [43]. The MRSV antiserum was tested on different natural hosts of the virus and was found to be applicable in immunodetection with root material indicating a systemic infection [10]. None of these antisera was shown to cross-react with another virus of this group, reflecting the insufficient aa sequence similarities of their P3 proteins. 

As most of the host plants infected with these novel plant RNA viruses are of high economical importance, specific and sensitive diagnostic tools are indispensable. Due to lack of sufficient sequence data for PPSMV and MRSV, primer pairs for RT-PCR, which are able to detect all RNA species and most of the existing isolates of these two putative viruses, are still rare. However, for the other four viruses, whose genomes are extensively sequenced, diagnostic primer pairs are available. Four diagnostic primer pairs were developed for EMARaV, which were shown to detect different isolates from Germany and Austria [14]. EMARaV RNA 3 was also detected by RT-PCR in single individuals of *Phytoptus pyri*, collected from galls on leaves of EMARaV infected mountain ash trees [79]. 

RT-PCR methods for FMV detection have been reported previously, amplifying parts of the RNAs carrying the N- and glycoprotein genes [5]. Meanwhile, primer pairs detecting all sequenced FMV RNAs were designed, which, for the first time, allowed a survey of the occurrence of FMV in countries from the African continent to Japan, just to mention a few [80,81,82,83,84]. Additionally, dot-spot-hybridization assays were successfully applied for FMV detection in fig seedlings, using probes synthesized from the positive strand of RNA 1 [6]. 

Primer pairs were developed for RRV that unambiguously detected RRV in 84 of 84 samples of cultivated roses and *R. multiflora*, showing typical symptoms of RRD, while RRV was not detected in 30 asymptomatic roses, used as negative controls [8]. RLBV-specific primer pairs detected all five RNAs of RLBV, not only in symptomatic field-grown raspberry plants, but also in experimentally infected *N. benthamiana* plants, in raspberry plants inoculated with viruliferous mites, and in RNA extracted from a bulked sample of mites collected from RLBD-affected plants [9].

Diagnostic primers are also available for the partially characterized PPSMV and MRSV [11,42]. Thus, in RT-PCR studies, PPSMV was sensitively detected in plants from different locations in India, as well as in pigeonpea experimentally infected by mites and grafting.

## 6. Virus Transmission

The diseases caused by RRV, RLBV FMV, PPSMV and MRSV were all shown to be transmitted by eriophyid mites, a group of arthropods which are very host specific and also serve as vector for several potyviruses [32,35,74,76,85,86]. This host specificity corresponds with the observation that just a few plant species are naturally infected by these viruses. 

**Figure 4:**
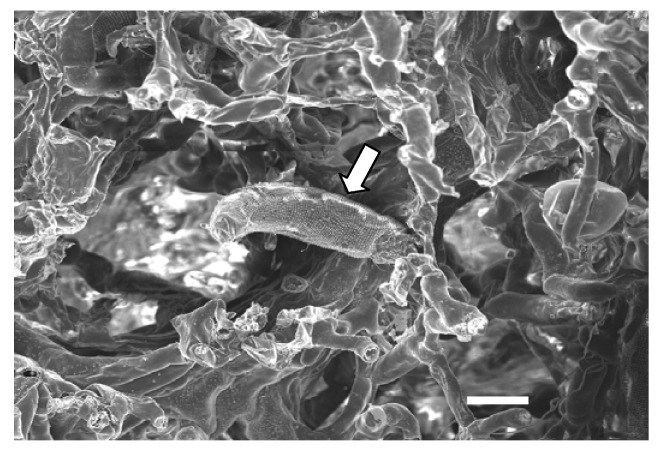
The eriophyid mite *Phytoptus pyri* (white arrow) within gall tissue from the undersurface of a leaf of an EMARaV infected mountain ash tree. Scanning electron microscopy. Bar represents 50 µm.

Only little information about the transmission mechanism of these eriophyid mites is available yet. Regarding EMARaV, vector transmission by an eriophyid mite species has not been shown convincingly so far. However, galls induced by the pear leaf blister mite *Phytoptus pyri* (*Eriophyidae*), are frequently found on EMARaV-infected *S. aucuparia* leaves (Figure 4). A detailed study using immune fluorescence microscopy and quantitative RT-PCR demonstrated that both the genomic and the complementary forms of RNA 3 of EMARaV and the putative nucleocapsid protein P3 are present inside the mite body [79], a finding which makes *Phytoptus pyri* a candidate vector for EMARaV.

The vector for fig mosaic disease is the free living eriophyid mite *Aceria ficus* Cotte [85,87]. Feeding by a single mite was demonstrated to be sufficient to transmit the virus to healthy seedlings of *F. carica *[87]. First disease symptoms, such as chlorotic spotting, mosaic and yellowing of the leaves, as well as malformation of the laminae, appeared approximately 40 days post infection (dpi). Infection was confirmed by the observation of virus-like particles in parenchyma cells by EM studies. However, it cannot be excluded that *A. ficus* is transmitting more than one virus, as FMD symptoms are discussed as being caused by mixed virus infections [4,5,21,25,26]. 

The wheat curl mite (*Aceria tosichella* Keifer, *Eriophyidae*) is the vector for both MRSV and WSMV, explaining the fact that a mixed infection with these two viruses is common. An investigation in Nebraska (USA) in 1995/96 detected both viruses in 40% of the collected samples [39]. The authors stated that the shared transmission by the same mite population could indicate a putative synergistic interaction between MRSV and WSMV. Both viruses are not only found in the same plant, but also in the same cell. Electron microscopy showed the different virus particles next to each other within wheat leaf cells [58]. Furthermore, the number of MRSV-associated virus-like particles was usually higher in these doubly infected cells than in cells without WSMV inclusions. HPD transmission by *A. tosichella* is restricted to feeding; the mite was shown to be unable to transmit transovarially [10,74]. 

*Aceria cajani* lives free on the undersurface of the leaves of pigeonpea and transmits PPSMV probably in a semi-persistent matter [76]. It is assumed that the virus does not replicate within the mite, which stays infectious for about 6–13 h. Under experimental conditions, pigeonpea plants showed first symptoms within 1–2 weeks after inoculation with mites collected from infected plants. Inoculation access period for PPSMV was at least 90 min and could be reduced by prior starvation to 60 min. The acquisition access period is at least 15 min. In these experiments, mites lost their ability for transmission 2–10 h after feeding on healthy plants. Here also, no transovarial transmission could be observed [76]. 

Due to their short stylet, eriophyid mites, living on the undersurface of plant leaves, may reach predominantly epidermal cells or the adjacent layers of the mesophyll. Thus, for effective virus transmission, these cell types must be infected. Indeed, virus-like particles associated with an infection of FMV, PPSMV or MRSV were shown to be present in parenchyma cells as well as in subepidermal leaf cells [21,50,58].

As eriophyid mites are highly host specific, their use for virus transmission on typical experimental host plants, e.g., *Chenopodium quinoa*, *Solanaceae *and *Cucurbitaceae*, for an improved feasible virus characterization is restricted. Without feeding on their host, they die quickly. Indeed, *Aceria cajani* was shown to survive only up to 13 h [76]. 

Interestingly, the putative mite vector itself may cause disease symptoms similar to virus infection. This was the case for the raspberry leaf blotch disorder, which was primarily reported to be only caused by the infestation with the raspberry leaf and bud mite *Phyllocoptes gracilis*, because no indication of a virus infection was found using conventional techniques, such as mechanical inoculation and grafting [35]. 

As vegetative transmission is typical for plant virus infection, PPSMV, FMV and EMARaV were demonstrated to be graft transmissible or could be passed by cuttings [11,88,89,90]. However, mechanical transmission of these virus-induced diseases was found to be difficult; none of the novel viruses could be passed by abrasion on its host plant so far. The only exception is MRSV, which could be transmitted mechanically by vascular puncture inoculation on maize ([59,75,77,91]. Inhibiting secondary metabolites as polyphenols or instability of the virus particles were discussed as basic causes [11]. Nevertheless, with some difficulties PPSMV and FMV could be transmitted mechanically on tobacco plants. *N. clevelandii* and *N. benthamiana* were infected by PPSMV, inoculating a 2% nicotine solution homogenate with a success of 10%–40% [11]. Symptoms appeared not until 40 dpi on apical leaves as vein chlorosis and necrotic spots. However, symptoms were diverse and virus titer remained low, even after two passages, but virus infection could be confirmed by RT-PCR with specific primer pairs. FMV was also shown to be transmissible on *Nicotiana *species (*N. benthamiana* and *N. clevelandii*) as well as on *Chenopodium sp*., *Datura stramonium* and *Vigna unguiculata* [21]. For MRSV, no experimental host, except barley, is known. 

Of the described viruses, no vertical transmission by seed or pollen has been reported so far. First efforts to detect EMARaV by highly specific and sensitive RT-PCR in plants grown from seed of infected mountain ash also gave no indication for seed transmissibility, although EMARaV is detectable in the seed itself (unpublished). FMV also repeatedly tested negative for seed transmission [22,89]. When pigeonpea seedlings derived from seed of PPSMV-infected plants were tested by ELISA, none of them contained the virus [92]. In the seed itself, PPSMV was only detectable in the coat, but not in the cotyledons. 

However, MRSV showed a very low seed transmission rate of 0.008% [91]. Only three seedlings out of 38,482 and from different lots showed clearly visible mosaic symptoms. Furthermore, they were found positive for virus infection in ELISA testing and feeding experiments on barley. The authors remarked that a high greenhouse temperature was chosen for a better symptom development (34 °C day, 21 °C at night). Therefore, it remained unclear if seed transmission will occur under natural field conditions. 

Transmission by soil has not been reported for any of these viruses so far.

## 7. Conclusions

The well characterized EMARaV, FMV, RRV and RLBV were shown to share many similarities, indicating a strong phylogenetic relationship, which suggests that also the latter two belong to the genus *Emaravirus*. Although extensive sequence information is still lacking for MRSV and PPSMV, their well-documented biological and structural properties also argue in favor of a relationship to emaraviruses. All six viruses have a segmented RNA genome of negative polarity, and recent sequence data analyses showed significant amino acid identities of some of the encoded proteins. Furthermore, they are all associated with the occurrence of DMBs, variable in size, which might represent the virions. Whereas vector transmission has not yet been conclusively shown for EMARaV, all the other viruses are vectored by eriophyid mites. Sequence comparison indicated that these viruses are related to the family *Bunyaviridae* and the genus *Tenuivirus*, but the collected data on virus morphology, genome organization, RNA and protein sequences, as well as biological properties, did not allow assigning them into existing families or genera. Therefore, the floating genus *Emaravirus* has been established with EMARaV and FMV as the first members, but RRV and RLBV are promising candidates for the next viruses to be assigned to this genus.

In addition to the six novel plant viruses discussed here, there remain still unassigned viruses or virus-like agents which might belong to the same group. Among them is a pathogen associated with Redbud yellow ringspot disease [93], which was shown to appear with DMBs and is also transmitted by eriophyid mites [94]. Leaves of affected redbud trees (*Cercis canadensis* L.) show bright yellow ringspots or blotches, and often necrotic spots. Furthermore, trees are characterized by many dead twigs and smaller branches. Very recently a virus with a genome organization highly similar to emaraviruses was shown to be associated with the disease, which was provisionally named Redbud yellow ringspot associated virus (RYRSaV) [95]. Furthermore, a disease with ringspot symptoms on the leaves of S*orbus scopulina* Greene (Greene’s mountain ash) was found in Alaska. Virus-like membrane-bound spherical particles could be observed and an approximately 32 kDA protein was consistently present in extracts from only symptomatic leaves. Although antiserum directed against the 35 kDa N-protein of EMARaV did not react in Western blot analyses with the enriched 32 kDa protein, circumstantial evidence, such as disease symptoms, association of the disease with gall forming eriophyid mites and particle morphology of the putative viral agent, suggest some relationship to emaraviruses [96]. It is therefore very likely that more unidentified pathogens, which belong to the new plant RNA virus genus *Emaravirus*, might be detected. 

Although in recent years we could collect new information about this novel genus, the genomic organization of emaraviruses and their biological interaction with host plants are far from understood. Finally, it has to be considered that in no case Koch’s postulates were convincingly fulfilled. This is certainly due to the fact that no local lesion hosts have been identified yet and that the isolation of infectious virus particles was not possible so far. However, the close association of the novel viruses with typical disease patterns, the presence of virus-like particles only in symptomatic tissue, and the close relation of vector, virus and disease in almost all cases strongly support the hypothesis that the novel emaraviruses EMARaV and FMV and the *Emaravirus* related viruses RRV, RLBV and probably also MRSV and PPSMV are the causes of the corresponding plant diseases. 
